# Chemical‐induced aseptic meningitis as a result of intrathecal hydromorphone therapy: Case report

**DOI:** 10.1002/ccr3.4599

**Published:** 2021-08-16

**Authors:** Sydney Willhite, Sangeeta Juloori

**Affiliations:** ^1^ Indiana University School of Medicine Indianapolis IN USA; ^2^ IU Health Southern Indiana Physicians Bloomington IN USA

**Keywords:** chemical‐induced aseptic meningitis, drug‐induced aseptic meningitis

## Abstract

Chemical meningitis, though rare, is a diagnosis of exclusion that must be considered in patients presenting with neurologic symptoms of undetermined cause. It is likely that any substance in contact with CSF can be the culprit.

## Graphical abstract

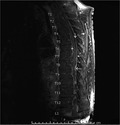

## 1 INTRODUCTION

Chemical meningitis is a rare, noninfectious complication of intrathecal manipulation. The common complaint of back pain may result in the need for neurosurgical procedures or placement of intrathecal pain pumps, which may give rise to this complication.

Chemical meningitis, a specific type of drug‐induced aseptic meningitis resulting from intrathecal injection, is a rare outcome that may be the result of drug solutions or other equipment utilized in neurosurgical procedures.^1^ It has been theorized to be caused by a hypersensitivity reaction or by direct meningeal irritation.^2^, ^3^, ^4^ It is characterized by lack of infectious etiology and by improvement in a few days without use of antibiotics. CSF (cerebrospinal fluid) analysis reveals pleocytosis typically of polymorphonuclear predominance but may be of lymphocytic or eosinophilic predominance as well; additionally, the CSF protein is usually elevated while the glucose level remains within normal limits.^1^

This report is of a patient diagnosed with chemical meningitis as a result of hydromorphone via an intrathecal pain pump who was diagnosed by process of exclusion based on CSF findings and cultures.

It is notable that this particular patient had an intrathecal pain pump placed at the T9 level by a neurosurgeon three years prior to this presentation. The pump is designed to last seven years and provides pain relief by way of constant and precise infusion of pain medication via a catheter.

## 2 CASE REPORT

An 81‐year‐old man with history of failed back syndrome warranting an intrathecal pain pump presented with sudden onset altered mental status (AMS) and fever reaching 38.3℃ of three days duration. He reported confusion one week prior lasting three days as well. Other complaints included difficultly initiating urination, chronic arthralgia, and an intermittent headache. Initial examination was notable for an exquisitely tender prostate, yet urinalysis showed no signs of infection. A complete blood count (CBC) at this time was within normal limits except for mild anemia, noted to be chronic, and for increased absolute eosinophils, which were attributed to seasonal allergies and were not a new finding. Ceftriaxone and acetaminophen were started while awaiting results of urine and blood cultures.

The following day, the patient was afebrile and his AMS had resolved. Additionally, neurology was consulted and had no concerns. The plan was to continue ceftriaxone while awaiting urine and blood cultures and to de‐escalate antibiotic treatment if no further signs of infection arose.

**FIGURE 1:**
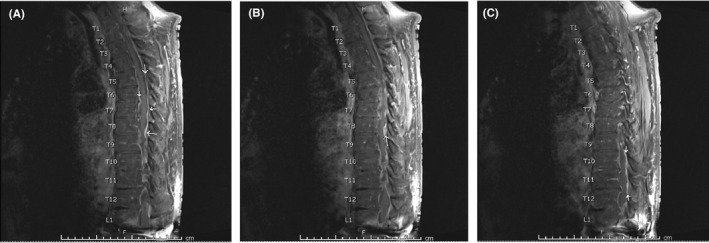
(A–C). MRI with contrast displaying abnormal meningeal enhancement in the mid‐ to lower thoracic spine

**FIGURE 2:**
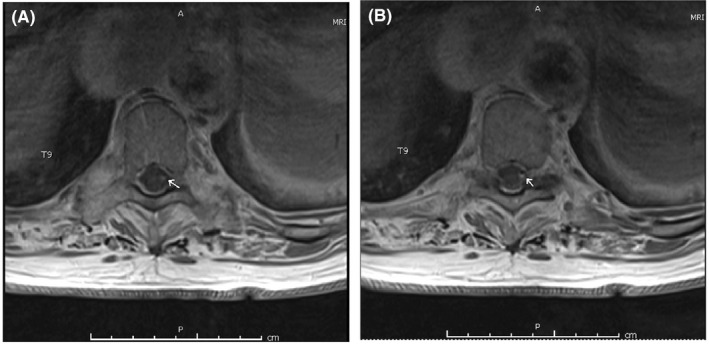
(A–B). MRI with contrast revealing question of focal contrast enhancement at the area in contact with the intrathecal pain pump catheter tip

On day three, the patient reported acute on chronic back pain and examination was notable for diffuse tenderness of the lower thoracic spine. A lumbar and thoracic MRI revealed meningeal enhancement of the mid to lower thoracic spine (Figure 1) and question of focal contrast enhancement in contact with the catheter tip (Figure 2). With concern for intrathecal pump catheter infection or chemical meningitis, a lumbar puncture was ordered, ceftriaxone was continued, and vancomycin and ampicillin were started. CSF results are shown in Table 1.

**TABLE 1:** CSF results revealing no infectious cause

CSF Cell Count/Differential		Reference Value
Color	Colorless	
Clarity	Clear	
Xanthochromia	Colorless	
Total nucleated cell count (TNC)	318/cumm (↑)	0–5
TNC Uncorrected	318/cumm (↑)	0–5
Red Blood Cell Count (RBC)	0/cumm	0–5
Bands/Neutrophils	15% (↑)	0%–2%
Lymphocytes	13% (↓)	63%–99%
Eosinophils	65% (↑)	0%
Basophils/Mast Cells	2%	
Monocytes/Macrophages	5%	3%–37%
Cells Cnt in Differential	100	
CSF Studies		
Glucose	42 mg/dl	40–70
Protein	135 mg/dl (↑)	15–45
Viral FS		
Varicella Zoster	Not detected	
Escherichia coli K1	Not detected	
Haemophilus influenza	Not detected	
Listeria monocytogenes	Not detected	
Neisseria meningitidis	Not detected	
Streptococcus agalactiae	Not detected	
Streptococcus pneumoniae	Not detected	
Cytomegalovirus	Not detected	
Enterovirus	Not detected	
Herpes simplex virus 1	Not detected	
Herpes simplex virus 2	Not detected	
Human herpesvirus 6	Not detected	
Human parechovirus	Not detected	
Varicella zoster virus	Not detected	
C. neoformans/gattii	Not detected	
CSF Culture		
Fungal Culture	No growth	

The CSF studies were most significant for eosinophilia and elevated CSF protein, making chemical meningitis caused by the hydromorphone via the intrathecal pump the most likely cause. No viral, bacterial, or fungal cause was found. The patient was started on dexamethasone and began improving. He was discharged after six days with no further symptoms. A dexamethasone taper began, and the patient followed up with his neurosurgeon. The hydromorphone was changed to fentanyl to prevent future chemical meningitis. This was the medication of choice as he had been intolerant of morphine via his intrathecal pump previously.

## 3 DISCUSSION

We diagnosed this patient with chemical meningitis after CSF analysis ruled out infectious etiology and instead revealed pleocytosis of eosinophilic predominance, protein three times the upper limit of normal, and glucose within the normal range.

As true chemical meningitis is rare, there are a limited number of reported cases due to direct intrathecal injection. Yet, it is believed that direct injection of any substance into the CSF could result in chemical meningitis.^2^ Such reactions have occurred with intrathecal use of baclofen,^2^ morphine,^5^ radiographic agents,^2^ anesthetic agents,^6^ aminoglycosides,^7^ and corticosteroids.^8^

Hydromorphone is a morphine derivative with a hydrogenated ketone that has an identical chemical formula and molecular weight as morphine.^9^, ^10^ As morphine and hydromorphone are very similar small molecules, it is likely that both can be the culprit of chemical meningitis; this has been previously demonstrated with intrathecal morphine.^5^ Additionally, as previously mentioned, it is believed that any molecule injected into the CSF can cause chemical meningitis likely via meningeal irritation.

Lastly, case reports have shown that intrathecal morphine can result in granuloma formation leading to spinal cord compression^11^ and experiments conducted in dogs resulted in similar granuloma formation with intrathecal morphine, hydromorphone, and fentanyl.^12^ It is suspected that histamine release occurs via mast cell degranulation and inflammatory cells subsequently exit the vasculature and form granulomas.^12^ This worrisome complication warrants imaging in cases of suspected chemical meningitis to ensure granuloma formation is not the direct cause of neurologic symptoms.

## 4 CONCLUSION

Chemical meningitis is a diagnosis of exclusion that must be considered in patients experiencing fever and neurologic symptoms who have had direct intrathecal injection. As it is believed that any molecule can be the cause, chemical meningitis must remain in the differential in a multitude of scenarios. This is of particular importance as pain pumps continue to be implanted for the large number of patients experiencing chronic pain.

## CONFLICT OF INTEREST

None.

## AUTHOR CONTRIBUTIONS

SW: Assisted in treatment of the patient and described the manuscript.

SJ: Treated the patient and revised the manuscript.

## ETHICAL APPROVAL

The patient provided consent for publication of this deidentified case.

## Data Availability

The authors confirm that the data supporting the findings of this case are available within the article, including tables and figures.
